# The ESICM datathon and the ESICM and ICMx data science strategy

**DOI:** 10.1186/s40635-024-00615-w

**Published:** 2024-03-12

**Authors:** Paul Elbers, Patrick Thoral, Lieuwe D. J. Bos, Massimiliano Greco, Pedro D. Wendel-Garcia, Ari Ercole

**Affiliations:** 1 Department of Intensive Care Medicine, Center for Critical Care Computational Intelligence (C4I), Amsterdam Medical Data Science (AMDS), Amsterdam Cardiovascular Science (ACS), Amsterdam Institute for Infection and Immunity (AII), Amsterdam Public Health (APH), Amsterdam UMC, Vrije Universiteit, Amsterdam, The Netherlands; 2 Department of Intensive Care Medicine, Amsterdam UMC, University of Amsterdam, Amsterdam, The Netherlands; 3 Department of Biomedical Sciences, Department of Anesthesiology and Intensive Care, Humanitas University, IRCCS Humanitas Research Hospital, Rozzano, Italy; 4 Institute of Intensive Care Medicine, University Hospital Zurich, Zurich, Switzerland; 5 Division of Anaesthesia and Cambridge Centre for AI in Medicine, University of Cambridge, Cambridge, UK

In this issue of Intensive Care Medicine Experimental we celebrate the success of the ESICM datathons. The fifth consecutive edition was held in the months of May and June, 2023, continuing the tradition of uniting teams of data scientists and intensive care professionals to spend up to 6 weeks to use large intensive care databases to take on clinically relevant challenges and provide new insights to benefit the intensive care community. We think you may agree with us that the quality of the results is impressive. But please do judge for yourself: abstracts of all finalists may be found in the electronic digital supplement.

Intensive Care Medicine Experimental considers data science, the field of study dedicated to the principled extraction of knowledge from complex data, as particularly relevant for the field of intensive care medicine, and as an important part of translational research [1]. In fact, Intensive care medicine is a natural habitat for data science and artificial intelligence [2]. Treating and caring for critically ill patients generates enormous amounts of data that are routinely collected and stored in electronic health records, more so than any other care episode. These data are not only voluminous but also diverse, coming from physiological monitoring, and life-support devices, clinical notes, medication/treatments, imaging and laboratory investigations. It is impossible for a human to completely appreciate and draw robust conclusions from such complexity. In addition, as intensive care providers, we must often deal with significant uncertainty related to diagnosis and prognosis. For each of our critically ill patients, we take many sequential decisions, often small, sometimes pivotal. The stakes are high: adverse events are always lurking around the corner with intensive care medicine having the highest rate of preventable harm [3], and many of our patients have adverse outcomes. In this context, it is easy to see how artificial intelligence models based on all previous critically ill patients could lead to bedside decision support to help intensive care professionals make better decisions to ultimately benefit future critically ill patients.

It is therefore hardly surprising that the ESICM Data Science Section has approved a strategy that continues to prioritize support for a so-called bytes-to-bedside approach to data science and artificial intelligence in intensive care medicine. Central to this approach is the long standing tradition in clinical medicine that each patient contributes to the learning experience of their healthcare professionals. This learning is extended with the datathon to the whole ICU community and beyond through the analysis of de-identified data. Imagine how data science and machine learning can facilitate this process at scale. The wisdom contained in the decisions made by millions of clinicians and the outcomes of billions of critically ill patients can and probably should inform the treatment and care of each future critically ill patient. As illustrated in the top half of Fig. 1, data related to intensive care treatments contribute to model development. These models should be integrated back in the electronic health record to provide decision support for intensive care professionals. And the resulting care and treatment should again inform new model development, thus closing the loop.

**Fig. 1:**
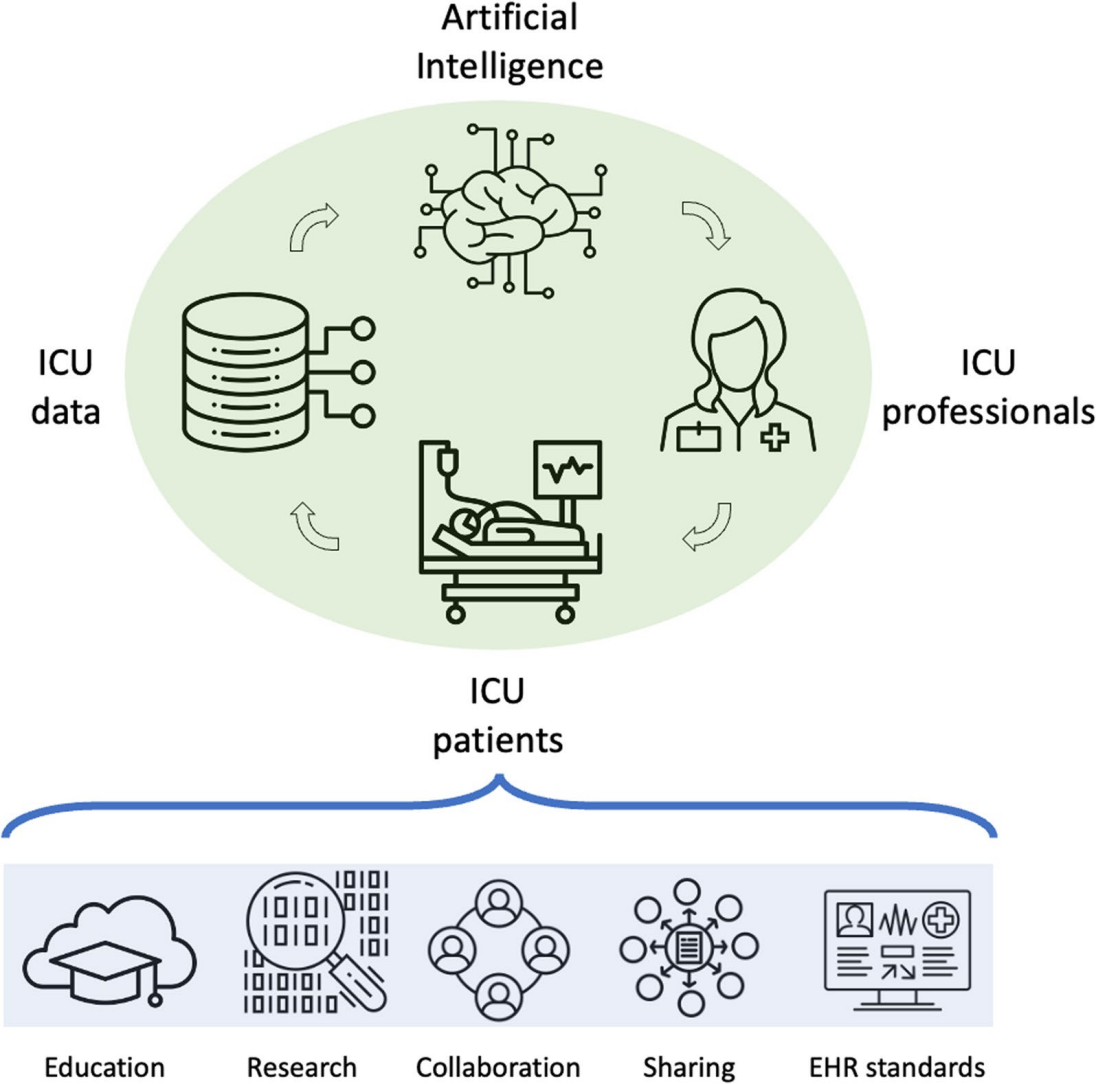
The circular bytes-to-bedside approach to data science and artificial intelligence: the care and treatment of critically ill patients generates large amounts of data that are routinely stored in electronic health records. These data may be leveraged to develop predictive models using artificial intelligence techniques. These models may be deployed as software that interacts with the electronic health record providing computerized decision support to intensive care professionals to improve care and treatment at the bedside of critically ill patients. The five pillars from the ESICM data science section strategy aim to support this circular bytes-to-bedside approach: providing data science education for intensive care professionals, promoting of data science research in intensive care medicine, providing guidance on responsible data sharing, benchmarking and standard setting for electronic health records in intensive care units and maintaining a framework for collaboration in the field of data science and intensive care medicine

The timeliness of such a strategy is probably best illustrated by the fact that only a handful of machine learning models have progressed to clinical implementation and even less to clinical evaluation of their effect on outcomes that are relevant to critically ill patients [4]. In fairness, the phenomenon of more papers than applications represents a translational gap that is far from unique to the field of artificial intelligence in intensive care medicine, but in the context of the possibly overhyped promise of machine learning in medicine that sparks the enormous scientific and societal interest, avoiding pitfalls and breaking down barriers toward bedside implementation is certainly commendable. Barriers include challenges related to large-scale data sharing for model development and validation, challenges related to compliance with regulations such as the EU medical device regulation and general data protection regulation as well as challenges related to integration of models in electronic health records.

As can be seen from the bottom half of Fig. 1, the bytes-to-bedside approach is supported by five pillars underpinning the ESICM Data Science section’s strategy: providing data science education for intensive care professionals, promoting of data science research in intensive care medicine, providing guidance on responsible data sharing, benchmarking and standard setting for electronic health records in intensive care units and maintaining a framework for collaboration in the field of data science and intensive care medicine. These five pillars are expected to lead to visible results in the form of concrete deliverables. These include the establishment of a data science course for intensive care professionals, support for data sharing initiatives, initiation of guidelines and systematic reviews at the crossroads of data science and intensive care medicine, mapping the implementation of electronic healthcare records across Europe, maintaining a database of data science contact persons for every intensive care unit in Europe and continue the success of the ESICM datathon.

The first two ESICM datathons were held together over a weekend in Milan, Italy under the inspiring leadership of ESICM past President Prof. Maurizio Cecconi alongside a series of lectures and seminars: the ESICM datatalk. During the COVID-19 pandemic, as society came to a halt and intensive care units were struggling, datathons continued with ESICM quickly converting them to an online format. This proved to be a paradigm shift. As well as attracting skills from other timezones and continents, the online format allowed the datathon to be held over the course of multiple weeks with review presentations along the way. Working remotely and using agile ‘sprint’ methodologies, this allowed teams of data scientists and intensive care professionals to make more progress and come up with astonishing achievements: the first two virtual datathons have already resulted in four peer-reviewed publications [5–8].

The challenges overcome here should not be underestimated. Data science research is inherently collaborative and requires a so-called programmatic approach which is very different from how trials or basic science is conducted. But the results show that these events do not only serve to introduce data science to intensivists and intensive care medicine to data scientists but may also contribute directly to discovery and new insights at the crossroads of these disciplines.

The 2023 edition of the ESICM datathon certainly continued the now well-established tradition of scientific quality. Organized in collaboration with Amsterdam UMC and using the first freely available European intensive care database, AmsterdamUMCdb [9], 12 teams took one of this year’s three challenges: mechanical power, transfusion and ICU capacity. After 4 weeks, there were still 11 teams competing for the top spots. During the Grand Final, a worldwide free live streaming event, the jury faced the task of selecting winners. As you may verify in the Additional file 1 containing all abstracts, this was a challenging task. After lengthy deliberation, the jury decided to award two special mentions and three prizes (Table 1). The top three teams presented their work in the Tech Forum at ESICM Lives 2023 Milan this last October.

**Table 1:** Winners and specials mentions (S) of the 2023 ESICM datathon

#	Team name	Team Leader	Country	Subject
1	The Vanguards	Anirban Bhattacharyya	USA	Mechanical power
2	Positive Pressure Posse	Laurens Biesheuvel	NL	Mechanical power
3	Medi-Terra-Neans	Lorenzo Querzi	IT	Capacity
S	Acid	Jan Goertzen-Patin	DE	Mechanical power
S	Bed Bytes	Romit Samanta	UK	Capacity

We hope to see you at the next edition of the datathon. Data scientists and intensive care professionals, unite! Together, we can improve intensive care!

### Supplementary Information

**Additional file 1.** Abstract Book, ESICM Datathon 2023.

## Data Availability

Not applicable.
