# Supplementary material for: The ESICM datathon and the ESICM and ICMx data science strategy
Source: Intensive Care Med Exp. 2024 Mar 12;12:29. doi: 10.1186/s40635-024-00615-w (PMC10933238; doi:10.1186/s40635-024-00615-w)
Supplement: Supplementary file 1 — Additional file 1. Abstract Book, ESICM Datathon 2023. [file 40635_2024_615_MOESM1_ESM.pdf]

ESICM
EUROPEAN SOCIETY OF INTENSIVE CARE MEDICINE

DATATHON 2023

Abstract Book

Team 1: Stat Squad

Heterogeneity in treatment effects of lower mechanical power

Itsuki Osawa[1], Tadahiro Goto[2], Takaya Nakashima[2], Mineto Fujisawa[3], Naoki Ito[2,3], Norikazu Hanioka[4], Sakina Kadomatsu[5], Raito Sato[4], Yoshihiko Raita[6], Junichiro Shibata[3], Kiyoyasu Fukuchi[3]

[1]Department of Emergency and Critical Care Medicine, University of Tokyo Hospital, Tokyo, Japan. [2]TXP Medical Co. Ltd. Tokyo, Japan. [3]University of Tokyo, Tokyo, Japan. [4]Medical Department, University of Fukui, Fukui, Japan. [5]Faculty of Medicine, International University of Health and Welfare, Otawara, Japan. [6]Okinawa Chubu Hospital, Uruma, Japan.

Correspondence: I. Osawa (ioosawa-tky@umin.ac.jp)

Introduction. There is currently significant interest in developing an optimal strategy to prevent ventilator-induced lung injuries (VILI) in mechanically ventilated patients [1, 2]. One emerging concept in this field is the amount of energy transferred from the ventilator to the respiratory system, known as mechanical power (MP) [3, 4]. However, due to the potential heterogeneity in treatment effects of lower MP and the challenges in achieving lower MP in some severely critically ill patients [5], little is known about specific patient subpopulations that may be expected to improve prognosis with lower MP through successful adjustment of ventilator settings. In this context, we aimed to identify the individual treatment effects (ITEs) of lower MP and the targeted subpopulations in which lower MP should be attempted as long as the patient's condition permits.

Methods. We retrospectively identified patients who were intubated for ≥48 hours and subsequently discharged from the ICU alive from the AmsterdamUMCdb. For patients with multiple ICU admissions, we only used data from their first admission. We estimated the MP of each patient based on the following equation using data for 48 hours after intubation [6, 7]:

$$MP = 0.098 \cdot RR \cdot \{V_{!} \cdot (P_{"\#\#"} + \Delta P_{\$\%\&,})\}$$

We first visualized the association between MP and the duration of mechanical ventilation using a locally weighted scatterplot smoother (Lowess) curve. Then, to evaluate the heterogeneous effects of lower MP (defined as less than the median MP value [< 18 J/min]) on the duration of mechanical ventilation, we used the machine learning-based survival causal forest model [8] and adjusted for the following confounders (e.g., patient demographics, sequential organ failure assessment (SOFA) score at ICU admission, vital signs, laboratory data, blood gas data [pH, PaO_2/FiO_2, $PaCO_2$], and lung compliance [tidal volume/driving pressure] at intubation). After stratifying all patients into quintiles of estimated ITEs, we estimated conditional average treatment effects (CATEs) in each quintile group to assess the heterogeneity in treatment effects of lower MP and compared the characteristics in each group to identify the candidate determinants of treatment effects. By applying the policytree algorithm [9], we statistically selected the best criteria for patient subpopulations that should aim to achieve lower MP.

Results. Among 2,114 patients eligible for analysis, we found a non-linear association of lower MP with shorter duration of mechanical ventilation (Figure 1). The survival causal forest model revealed heterogeneity in the treatment effect of lower MP (Figure 2A). The policytree algorithm statistically identified the potential target subpopulations of lower MP as patients with prominent lower lung compliance (lung compliance < 38 mL/cmH_2O) or those not-requiring high positive end-expiratory pressure (PEEP < 10 cmH_2O) (Figure 2B). Lower MP in the target subpopulation was significantly associated with shorter duration of mechanical ventilation (-1.5 days [95%CI -2.1 days to -0.6 days]) compared to higher MP. We verified that 46% of the target population could achieve lower MP by permitting hypercapnia.

Conclusions. Patients with low lung compliance or those not-requiring high PEEP would benefit most from achieving lower MP.

References

1. Tremblay LN, Slutsky AS. Ventilator-induced lung injury: from the bench to the bedside. Intensive Care Med. 2006;32(1):24-33.
2. Slutsky AS, Ranieri VM. Ventilator-induced lung injury [published correction appears in N Engl J Med. 2014 Apr 24;370(17):1668-9]. N Engl J Med. 2013;369(22):2126-2136.
3. Gattinoni L, Tonetti T, Cressoni M, et al. Ventilator-related causes of lung injury: the mechanical power. Intensive Care Med. 2016;42(10):1567-1575.
4. Serpa Neto A, Deliberato RO, Johnson AEW, et al. Mechanical power of ventilation is associated with mortality in critically ill patients: an analysis of patients in two observational cohorts. Intensive Care Med. 2018;44(11):1914-1922.
5. Pelosi P, Ball L, Barbas CSV, et al. Personalized mechanical ventilation in acute respiratory distress syndrome. Crit Care. 2021;25(1):250.
6. Becher T, van der Staay M, Schädler D, Frerichs I, Weiler N. Calculation of mechanical power for pressure-controlled ventilation. Intensive Care Med. 2019;45(9):1321-1323.
7. Trinkle CA, Broaddus RN, Sturgill JL, Waters CM, Morris PE. Simple, accurate calculation of mechanical power in pressure controlled ventilation (PCV). Intensive Care Med Exp. 2022;10(1):22.
8. Cui Y, Kosorok MR, Sverdup E, Wager S, Zhu Ruoqing. Estimating Heterogeneous Treatment Effects with Right-Censored Data via Causal Survival Forests. Journal of the Royal Statistical Society Series B: Statistical Methodology. 2023;85(2):179-211.
9. Athey S, Wager S. Policy learning with observational data. Econometrica. 2021;89(1):133-161.

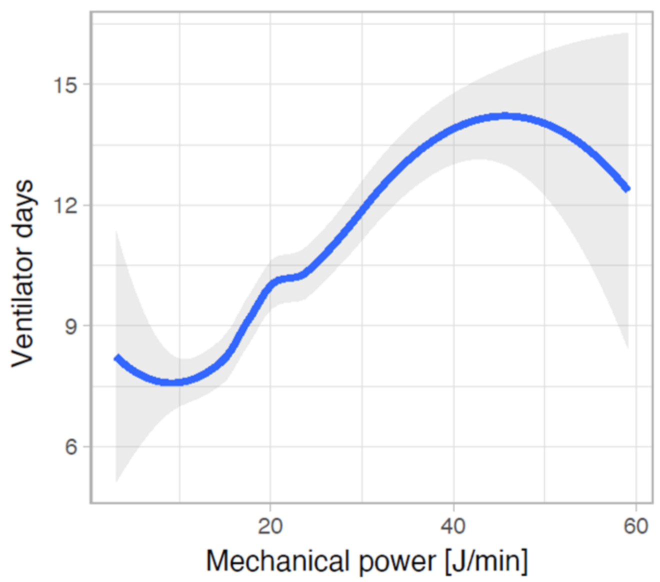

Figure 1. Association between MP and the duration of mechanical ventilation using a locally weighted scatterplot smoother (Lowess) curve.

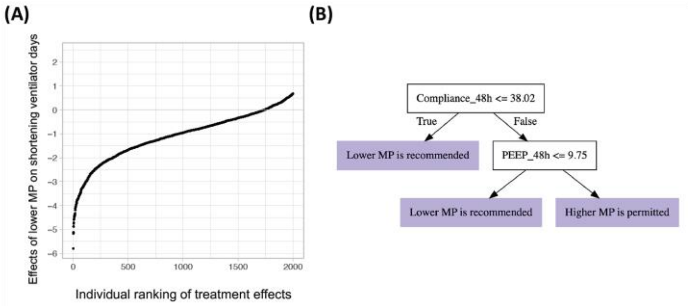

Figure 2. Heterogeneity in treatment effects of lower MP and its potential target subpopulations.

Team 2: Datathon Deserts

Deciphering Mechanical Power Components: An XGBoost Analysis of Factors Contributing to the Progression of Lung Injury

Lada Lijović[1,2], Zheng Feng[1], Fatime Hawchar[3], Ivaylo Minev[4], Stipe Pelajić[2], Beatriz Helena Cermaria Soares da Silva[5,6], Nedelcho V. Stoykov[7], Ana Marija Štrucl[8,9], Alessandra Angelucci[10]

[1]Amsterdam UMC, Vrije Universiteit, Department of Intensive Care Medicine, Laboratory for Critical Care Computational Intelligence, Medical Data Science, Public Health, Cardiovascular Science, Institute for Infection and Immunity, Amsterdam, Netherlands. [2]University Hospital Center Sestre Milosrdnice, Department of Anesthesiology, Intensive Care and Pain Management, Zagreb, Croatia. [3]University of Szeged, Department of Anesthesiology and Intensive Care, Szeged, Hungary. [4]Medical University of Plovdiv, University Hospital St. George, Department of Anaesthesiology, Emergency and Intensive Care Medicine, CC PERIMED, Plovdiv, Bulgaria, [5]Universidade Nove de Julho, Diretoria de Ciências Médicas, Campus Guarulhos, São Paulo, Brazil, [6]Universidade Federal de São Paulo, Departamento de Anestesiologia, Dor e Terapia Intensiva, São Paulo, Brazil, [7]Technical University of Sofia - Plovdiv Branch, Plovdiv, Bulgaria, [8]Gynaecology and Obstetrics Hospital Kranj, Kranj, Slovenia, [9]University of Ljubljana, Faculty of Medicine, Ljubljana, Slovenia, [10]Politecnico di Milano, Dipartimento di Elettronica, Informazione e Bioingegneria, Milano, Italy

Correspondence: F. Hawchar (fatimehawchar@gmail.com)

Introduction. Mechanical power (MP) has been increasingly recognized as the cause of ventilator induced lung injury (VILI). The original paper by Gattinoni suggested that not all MP components contribute equally to the resulting power applied. It is unknown which parameters of MP contribute to VILI the most. Furthermore, it omits the concept of duration of application of the components.

Methods. A retrospective study evaluating mechanically ventilated patients from AmsterdamUMC ICU Database was performed. To exclude patient contribution to work of breathing, patients ventilated in volume controlled and/or pressure controlled modes were selected. For each patient, parameters suggested as contributors to MP calculation were derived: tidal volume adjusted for predicted body weight and dead space by Harris Benedict formula, driving pressure, respiratory rate, inspiratory time, I:E ratio, and flow. As compliance and resistance were not available in the database, and are derived values from parameters already used in the calculation of MP, they were excluded. To modify for confounding factors, age and initial SOFA score were added as predictors. Duration of application of each component was used as a predicting variable, as well as total time of mechanical ventilation applied until outcome measurement. An Extreme Gradient Boosting model (XGBoost) was firstly built to predict lung injury severity inferred from PaO_2/FiO_2 ratio and PEEP, aiming to ensure that the scoring method was robust and there was strong association between predictors and lung injury severity. The second XGboost classification model was built for predicting worsening or improving lung function based on lung injury severity score. Different weights were assigned to each measurement based on the frequency of the class the lung

injury change belongs to. Dataset was partitioned into distinct training, validation, and testing segments with 64%, 16%, and 20% allotments respectively. Results

Results. A total 112012 hours of mechanical ventilation, pertaining to 1888 patients, were analysed. The model exhibited an accuracy of 0.90 on the training dataset, demonstrating its capacity to capture underlying patterns effectively. Upon validation, the model yielded an accuracy of 0.84, indicating its generalizability. On the unseen test data, the model's accuracy remained at 0.83, supporting its predictive power. Macro average one vs. rest AUC-ROC score for the test dataset was found to be 0.711, showing a balanced trade-off between sensitivity and specificity. One vs. rest for class getting_worse AUC-ROC was 0.77 (Figure 1). Most important feature measured by gain importance is time spent in mechanical ventilation and duration of application of a component up to lung damage worsening moment, followed by adjusted tidal volume (Figure 2).

Conclusion. Our model highlighted the duration of mechanical ventilation as a critical feature and duration of each component applied, followed by normalized tidal volume. Temporal exposure to these factors may play a more decisive role than individual values of each parameter. Future research should, besides including more confounders, analyse a more extensive dataset, particularly one featuring a higher number of ARDS patients, longer ventilation periods and the availability of ventilation curves for direct MP assessment.

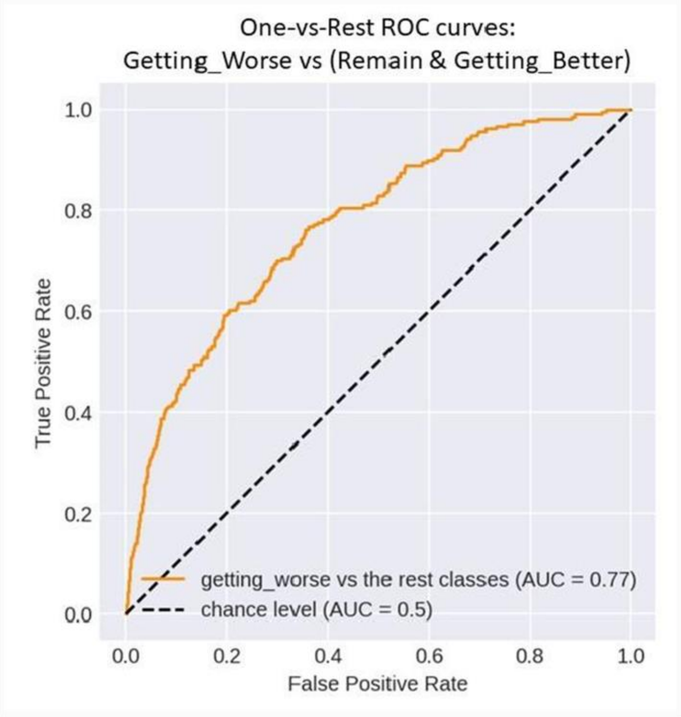

Figure 1. One-vs-Rest ROC curve of the XGBoost Model illustrates the performance of our XGBoost model for multi-class classification using a one-vs-rest strategy. (Getting_worse means worsening of lung injury, compared to the rest classes: lung injury improvement and unchanged function).

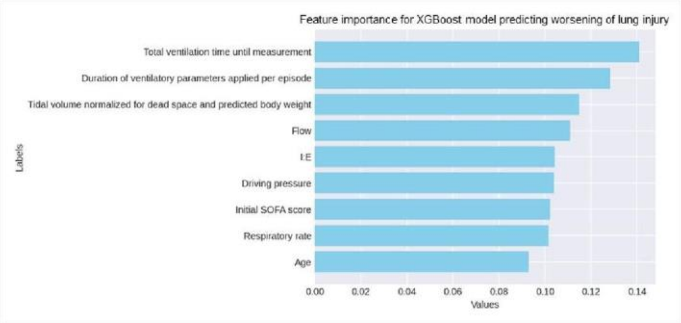

Figure 2. Bar chart illustrating the relative importance of each feature in the prediction task (worsening of lung injury) of XGBoost model.

Team 3: Medi-Terra-Neans

Modelling Variability of Intensive Care Unit Demand: A Comparative Cost Analysis and Performance Evaluation of Fixed versus Variable Beds Arrangements with a simulation approach.

Lorenzo Querci[1,2], Orlando Sagliocco[3], Berrin Er[4], Francesca Mulazzani[5], Beatrice Brunoni[5], Aydogan Arslantas[6], Mustafa Kemal Arslantas[7]

[1]Anesthesia and Intensive Care Resident at University of Bologna, Italy. [2]Grande Ospedale Metropolitano Niguarda Ca' Grande, Milan, Italy. [3]Intensive Care Unit, ASST Bergamo Est, Italy. [4]University of Health Sciences, Ankara Bilkent City Hospital, Turkey. [5]Anesthesia and Intensive Care Resident at University of Milano-Bicocca, Italy. [6]Fenerbahce University, Istanbul, Turkey. [7]Demioglu Bilim University, Istanbul, Turkey

Correspondence: L. Querci (querci.lorenzo@gmail.com)

Introduction. High demand, limited intensive care unit (ICU) beds, and costs pose a sustainability challenge in healthcare. Simulation modelling helps examine ICU bed assignment policies [1]. Estimating length of stay (LOS) is a complex process [2, 3] due to ICU daily uncertainties. This study aimed to compare the effectiveness of two management strategies: fixed versus variable bed arrangements. We developed an integrated ICU LOS prediction model incorporated within a comprehensive simulation model.

Methods. Anonymised data of adult patients were used from AmsterdamUMCdb (2003-2016) [4]. We proposed a statistical approach based on Monte Carlo Simulation (MCS) to determine the optimal number of beds in an ICU. Our customised simulation model was developed to assess the number of patients rejected from the ICU due to total capacity and the hours the beds remain available. Admission rate (AR) was estimated from elective and urgent patients' cumulative AR. Using different coefficients for four-time daily slots, days of the week and months, we estimated the lambda parameter of a Poisson distribution and simulated hourly random admissions for five scenarios in a realistic fashion. The simulation was based on an algorithm (Figure 1) which contains the patient management from the request of ICU admission to discharge. LOS of admitted patients was estimated by stochastic bootstrapping [5, 6]. In the variable-bed simulation, a probabilistic approach every 24 hours for just admitted patients (stochastic or random forest regression-based), the estimation of incoming patients for the next seven days and a stochastic estimation of LOS for these allowed for the opening or closing of variable beds seven days in advance within a range of 0.875 to 1.125 times the calculated ideal fixed-bed capacity. The best fixed-bed ICU number and the best cut-off for the probability of needing open/closed beds were obtained by minimising costs. In a closed system, a patient unsuccessfully demanding ICU was considered rejected. According to the literature [7, 8], we estimate five different costs for rejected patients and beds free. The primary study endpoint was to describe the cost difference between fixed beds, stochastic-variable beds, and prediction-based-variable beds ICU.

Results. In the first scenario simulated, using the stock of the first pair of costs (€612.75 for 24-hours available bed and €21927.22 for an ICU rejected patient), the best fixed-bed ICU capacity was 38 beds (Figure 2A), with a total cost of €8746±1377. In the same condition, the stochastic variable-bed approach, with the best cut-off for open/closing probability about 0.9, obtains €9033±2476 (Figure 2B). The random forest regression model showed a testing correlation from 0.39 to 0.49 using variables available from day one to days one to five. Variable bed policy proves cost-effective in almost all scenarios independently from the prediction method (Figure 2C).

Conclusion. This study presents a novel approach to identifying and addressing ICU bed shortages by implementing variable beds. Our simulation results demonstrate that a variable-bed ICU model enables effective resource allocation planning.

Further studies will be needed to define the feasibility and performance of ICUs with variable bed arrangements.

References

1. Kusum S. Mathews, Elisa F. Long (2015) A Conceptual Framework for Improving Critical Care Patient Flow and Bed Use. Ann Am Thorac Soc 12:886–894.
2. Verburg et al. (2017) Which Models Can I Use to Predict Adult ICU Length of Stay? A Systematic Review. Crit Care Med.
3. Bahalkeh E, Hasan I, Yih Y (2022) The relationship between Intensive Care Unit Length of Stay information and its operational performance. Healthc Anal 2:100036.
4. Thoral PJ, Peppink JM, Driessen RH, et al (2021) Sharing ICU Patient Data Responsibly Under the Society of Critical Care Medicine/European Society of Intensive Care Medicine Joint Data Science Collaboration: The Amsterdam University Medical Centers Database (AmsterdamUMCdb) Example*. Crit Care Med 49:e563.
5. Bai J, Fügener A, Schoenfelder J, Brunner JO (2018) Operations research in intensive care unit management: a literature review. Health Care Manag Sci 21:1–24.
6. Bai J, Fügener A, Gönsch J, et al (2021) Managing admission and discharge processes in intensive care units. Health Care Manag Sci 24:666–685.
7. Edbrooke DL, Minelli C, Mills GH, et al (2011) Implications of ICU triage decisions on patient mortality: a cost-effectiveness analysis. Crit Care 15:R56.
8. Lefrant J-Y, Garrigues B, Pribil C, et al (2015) The daily cost of ICU patients: A micro-costing study in 23 French Intensive Care Units. Anaesth Crit Care Pain Med 34:151–157.

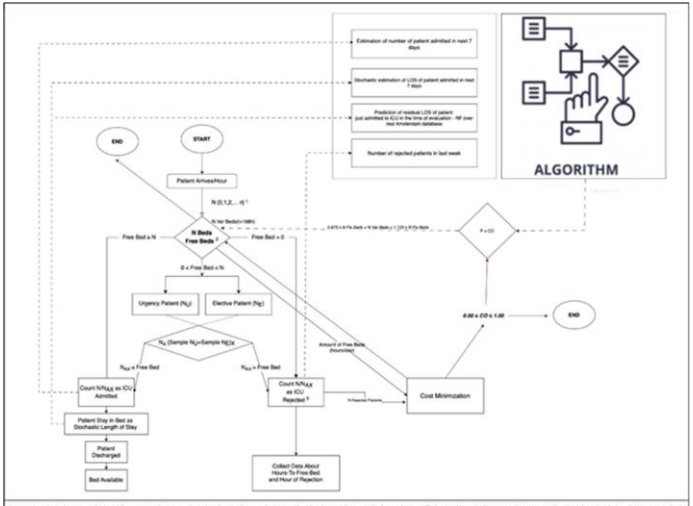

1: Hourly admission rate derived from a random number simulation from a Poisson distribution with Lambda from the coefficient table. 2: The counting starts after 720 hours of simulation to exclude the fact that in the simulation, the intensive care unit starts with all beds empty. 3: Dayilly number of patients rehected

Figure 1. The algorithm considers an admission rate based on an hourly probability distribution following a Poisson distribution with lambda derived from the array of admission coefficients. Patients requesting admission may be accepted or rejected depending on the availability of beds. If the available beds cannot accommodate all patients, those with urgent admission types are prioritised and accepted first. The LOS is modelled stochastically by bootstrapping from the LOS distribution in the Amsterdam UMC database [4]. The first 30 simulation days were not considered in the result, considering that ICU started with empty beds. The simulation tested beds ranged from 2 to 60, with a simulation duration of 12 months conventionally of 28 days each, for 8448 hours, and a simulation number set to S = 100. Each simulation was repeated for five ICU scenarios using cumulative admission rates, hours-days-months coefficients, normal error, or multiplicative coefficients. A complex simulation that incorporates optimal variation in the number of beds within a range of 0.875 to 1.125 times the calculated ideal bed capacity was built. A probability score executed every 24 hours allowed for the opening or closing of variable beds seven days in advance. The prediction model was based on an MC simulation that considered the estimation of remaining LOS for admitted patients (stochastic or prediction based on patients' variables), the estimation of incoming patients for the next seven days, and a stochastic estimation of LOS for these patients utilising the available simulation information at the time of prediction. In addition to the above factors, other variables are considered to assess the presence of rejected patients in the previous three days and the presence of significantly different coefficients on consecutive days. These variables help evaluate the historical trend of rejected admissions and identify any patterns or anomalies in the probability over consecutive days.

Another MC simulation was used to optimise this probability cut-off to minimise costs.

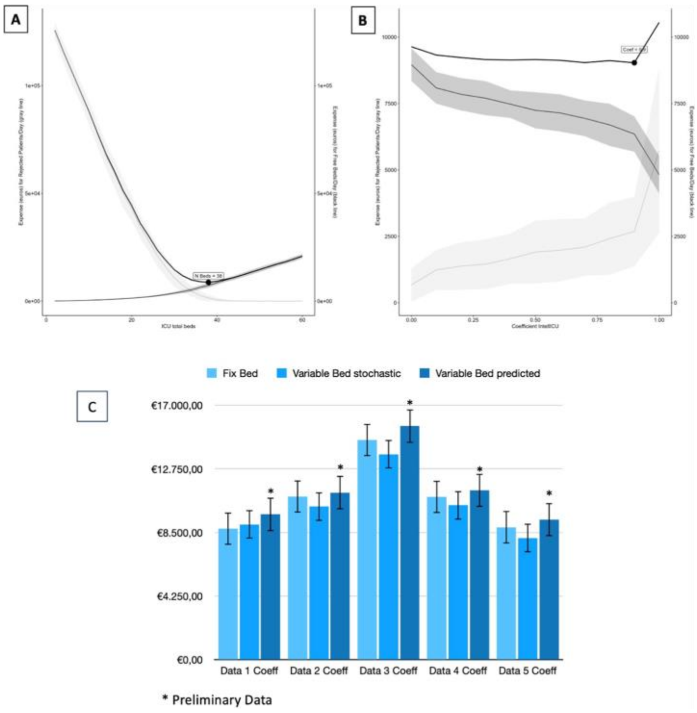

Figure 2. (A) Simulation to find the best number of beds minimising costs for rejected patients and unused beds. The simulation considers several beds ranging from 2 to 60, a simulation duration of 12 months, and 100 simulations. Thirty-eight beds represent the minimisation of costs for unused beds and rejected patients. **(B)** Simulation to find the best cut-off for the probability of needing an opening/closing bed starting in a 38 beds ICU. The simulation considers a coefficient from 0.0 to 1, a simulation duration of 12 months and 100 simulations. A coefficient of about 0.9 permits minimising the sum of costs for unused beds and rejected patients. **(C)** Different admission rate scenarios compare bed arrangement policies and prediction methods for fixed and variable bed policies (stochastic and random forest). Variable bed policy proves cost-effective in almost all scenarios independently from the prediction method—preliminary data.

ESICM Datathon 2023 Winner

Team 4: The Vanguards

The Power of Causal Inference: Unraveling the Link Between Mechanical Power and Patient Outcomes

Anirban Bhattacharyya[1], Arif Canakoglu[2], Markos Kashiouris[3], Anya Mathur[4], Piyush Mathur[5], Ronit Nath[6], Jacob Vine[7], Ken Koon Wong[5], Chaoping Wu[5]

[1]Mayo Clinic, Rochester, USA. [2]Policlinico di Milano, Milano, Italy. [3]Inova Fairfax Hospital, Falls Church, USA. [4]Western Reserve Academy, Hudson, USA. [5]Cleveland Clinic, Cleveland, USA. [6]University of California, Berkley, USA. [7]Beth Israel Deaconess Medical Center, Boston, USA.

Correspondence: A. Bhattacharyya (anirbanb007@gmail.com)

Introduction. Mechanical power (MP) combines various factors contributing to ventilator-induced lung injury (VILI) into a single measure, assessing the energy exerted on the lung by the ventilator [1]. Understanding the implications of MP on different disease processes can aid in the development of safer ventilation strategies. Although numerous studies have demonstrated an association between MP and VILI-related outcomes, limited evidence exists regarding the causal relationship. Hence, we aim to establish the causal effect of MP and quantitatively measure its impact on clinical outcomes through the utilization of causal inference methods in various cohorts.

Methods. We conducted a retrospective cohort study on patients with invasive mechanical ventilation (IMV) using de-identified data from AmsterdamUMCdb. To address the potential confounding variables, a directed acyclic graph (DAG) was constructed. A systematic approach utilizing the estimands was employed, employing backdoor adjustment and the do operator, to estimate the causal effect of MP. In accordance with the DAG, the study focused on 28-day ventilator-free days (VFDs) and the SpO_2/FiO_2 ratio (SFR) as primary outcomes [2]. The primary objective of initial analysis is estimating the average treatment effect (ATE) of MP on diverse patient cohorts' outcomes, followed by the application of a dynamic model that incorporates time series data to further refine the ATE estimation of MP. To estimate an optimal MP threshold, we leveraged dynamic hourly database to train machine learning (ML) models at various MP levels. Causal inference was established through the utilization of the doWhy package and econML.

Results. Of the 13,558 unique admissions included in the Analysis 59.9% (7,997) were attributed to surgical reasons. The median intubation durations were 8 hours for surgical admissions and 65 hours for medical admissions. The median MP values were 11.7 J/min in the surgical population, while 16.1 J/min in the medical population. In analysis of VFDs, we observed lower ATEs (higher impact) of MP on the medical population compared to the surgical population, with estimated values of -0.16 (95% CI: -0.25 to -0.08) and -0.06 (-0.1 to -0.02) respectively. In the causal analysis of SFR within the first 48 hours of admission, the estimated ATEs of MP on the medical population were -1.26 (-3.17 to 0.65), whereas the estimates for the surgical population were -0.70 (-3.63 to 2.23). Our findings indicated a higher impact of MP on SFR in the medical population compared to the surgical population. (Figure 1). In 8,640 models trained with dynamic hourly database, we observed distinctive ATE patterns of MP between cohorts (Figure 2). Finally, to simulate the effect of MP, we created a online tool to estimate and visualize hourly ATE in medical and surgical population [3].

Conclusions. Using robust causal models, we successfully demonstrated that an increase in MP leads to poorer clinical outcomes in both 28-day VFDs and SFR. Furthermore, the impact of MP was found to be more pronounced in the medical population compared to the surgical population.

References

1. Gattinoni, L., T. Tonetti, M. Cressoni, P. Cadringher, P. Herrmann, O. Moerer, A. Protti et al. "Ventilator-related causes of lung injury: the mechanical power." Intensive care medicine 42 (2016): 1567-1575.

2. Kiciman, Emre and Dillon, Eleanor and Edge, Darren and Foster, Adam and Hilmkil, Agrin and Jennings, Joel and Ma, Chao and Ness, Robert Osazuwa and Pawlowski, Nick and Sharma, Amit and Zhang, Cheng A Causal AI Suite for Decision-Making NeurIPS 2022 Workshop on Causality for Real-world Impact, December 2022
3. https://kenkoonwong.shinyapps.io/team4_vanguard_mp_sim/

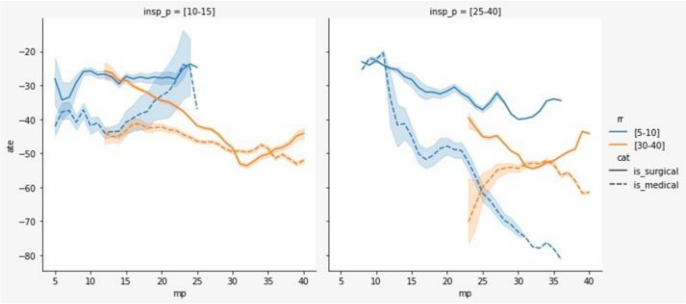

Figure 1. The relationship between mechanical power (mp) and average treatment effect (ate) in various respiratory rate (rr) categories, stratified by driving pressure (insp_p), for both the medical and surgical cohorts (solid and dashed line).

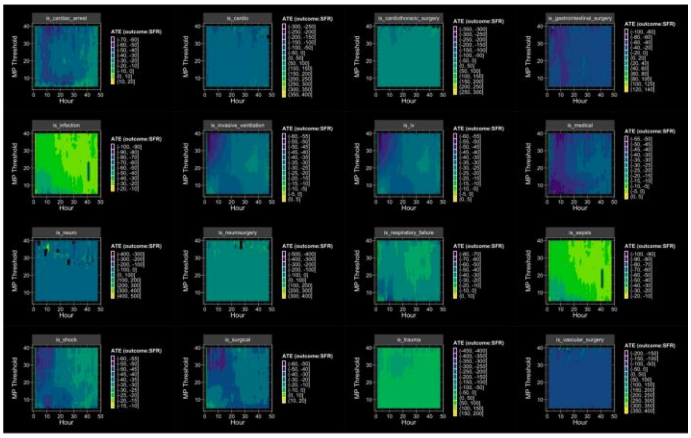

Figure 2. Illustrate distinctive patterns of mechanical power (MP) threshold in various patient population cohorts. The figure depicts the relationship between hours after intubation, MP threshold, and Average Treatment Effect (ATE), stratified by the respective patient population cohorts. In the heatmap, each color gradient represents a specific cohort, as indicated by the corresponding color legend.

Team 5: VentilAi

Enhancing ICU survival by individualizing mechanical power in mechanically ventilated patients

Kenny Rumindo[1], Ahmed S. Alkhalifah[2], Florian Blanchard[3], Johan Helleberg[4], Edgar Brincat[5,6], David Clarke[7], Benjamin Popoff[8], Olivier Duranteau[9], Zubair Umer Mohamed[10], Abdelrahman Senosy[11]

[1]Research & Development, Getinge Acute Care Therapies, Solna, Sweden. [2]Pediatric Critical Care unit, Qatif Central hospital, Dammam, Saudi Arabia. [3]Sorbonne University, GRC 29, AP-HP, DMU DREAM, Department of Anesthesiology and Critical Care, Pitié-Salpêtrière Hôpital, Paris, France. [4]Department of Perioperative Medicine and Intensive Care, Karolinska University Hospital, Stockholm, Sweden. [5]Paediatric Intensive Care Unit, Royal Hospital for Children, Glasgow, United Kingdom. [6]School of Medicine, Dentistry & Nursing, University of Glasgow. [7]Oxford Critical Care, Oxford University Healthcare Trust, Oxford, UK. [8]Department of Anesthesiology and Critical Care, Rouen University Hospital, Rouen, France. [9]Intensive care unit, Hôpital d'instruction des armées Percy, Clamart, France. [10]Adult Critical Care Unit, King Faisal Specialist Hospital and Research Centre, Madinah, Saudi Arabia. [11]Adult Intensive Care Unit, Hayat National Hospital, Medina, Saudi Arabia

Correspondence: E. Brincat (Edgar.Brincat@ggc.scot.nhs.uk)

Introduction. High mechanical power (MP) is correlated to in-hospital mortality in mechanically ventilated patients [1]. However, safe MP thresholds are not well studied [2]. Our primary objective was to assess the impact of individualized MP thresholds on ICU mortality. Secondary objective was to propose a method to individualize mechanical ventilation (MV) settings based on MP and other covariables.

Methods. This is a retrospective study on Amsterdam UMC database [3]. Inclusion criteria are patients older than 16 years old, requiring at least 48 hours (48h) of pressure-controlled MV. Exclusion criteria are death or extubation within 48h, and insufficient data. To find the safe MP thresholds, patients were grouped based on PaO_2/FiO_2 ratio (PFR) over the first 6 hours of MV (non-hypoxemic: PFR>300, mild: PFR=200-300, moderate: PFR=100-200 and severe hypoxemia PFR<100). Time-weighted average (TWA) MP over the first 48h was calculated and normalized to ideal body weight (IBW) [4]. To predict mortality, different machine learning models were evaluated using the area under the Receiver Operating Characteristic (ROC) curve (AUC). The individualization of MV settings were done in 2 steps: 1) PEEP was optimized using PEEP titration [5] to achieve the highest dynamic compliance; and 2) tidal volume or driving pressure and respiratory rate (RR) were optimized based on a predicted mortality and acidosis status [6]. A case study on the proposed individualization method was then performed on non-survivor patients.

Results. 2338 patients were analyzed and stratified into non-hypoxemic (615), mild (736), moderate (840), and severe hypoxemia (147). TWA-MP was higher among non-survivors compared to survivors (p<0.001). Unadjusted for IBW, TWA-MP was associated with ICU mortality with an odds ratio (OR) of 1.02, 95%CI (1.01 - 1.03) per 1 J/min increase. The upper limit for all patients was 16.51 J/min. Safe upper limits of IBW-adjusted MP (J/min/kg) were found to be 0.22 (non-hypoxemic), 0.27 (mild hypoxemia), and 0.34 (moderate hypoxemia). Statistically significant upper limit was not found for severely hypoxemic patients. Figure 1 displays COX-survival analysis using the upper limits and showed no significant

influence of 48h-TWA-MP on mortality (p=0.35). XGBoost performed best in predicting ICU mortality (AUROC=0.88). The IBW-adjusted 48h-TWA-MP has a limited effect in comparison to other covariables, as shown in the SHapley Additive exPlanations (SHAP) values in figure 2. The individualization method resulted in lower predicted mortality (58 survivors out of initially 614 non-survivors [9.4%]). In acidosis patients, the individualization method resulted in slightly lower PEEP and higher ventilation minute, while in non-acidosis patients it resulted in a higher PEEP and minimized MP.

Conclusion. This study identified safe upper limits of normalized MP in multiple hypoxemia groups except the severely hypoxemic group - potentially due to small sample size, missing data, comorbidities and varying pathologies. Individualized optimization of ventilator settings has the potential to improve patient outcomes. The proposed method of MV individualization showed promising results, but requires further investigations and verifications.

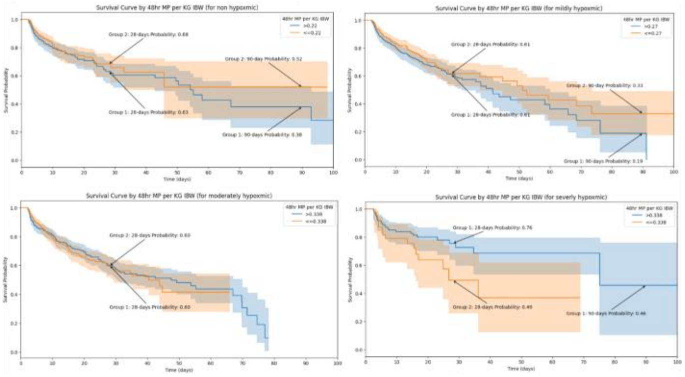

Figure 1. COX-survival curve according to the found safe limits of 48h-TWA, IBW-adjusted MP. Stratified into hypoxemic levels: non-hypoxemic (top left), mild hypoxemic (top right), moderate hypoxemic (bottom left), severe hypoxemic (bottom right).

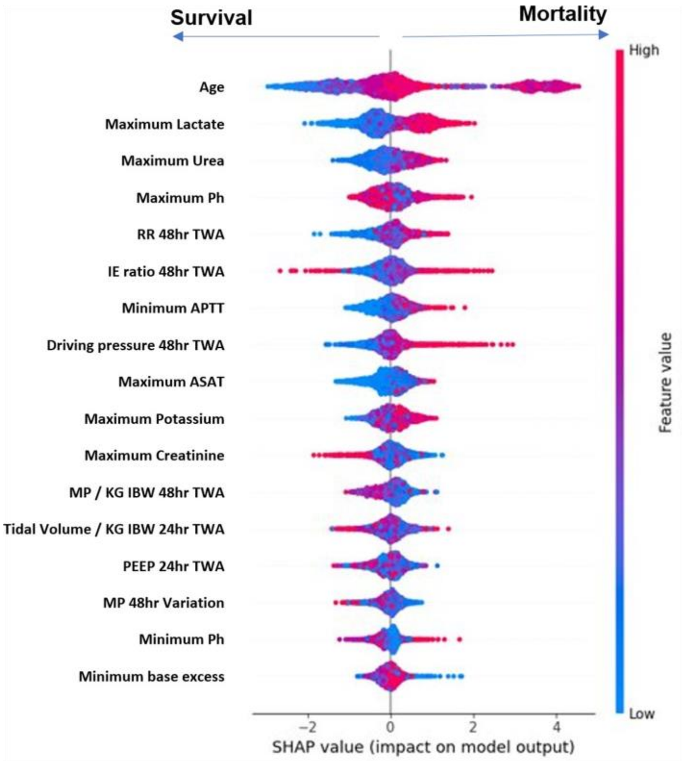

Figure 2. SHAP values displaying sensitivity of the XGBoost model in predicting ICU mortality against different covariables.

References

1. Neto AS, Deliberato RO, Johnson AEW, et al (2018) Mechanical power of ventilation is associated with mortality in critically ill patients: an analysis of patients in two observational cohorts. Intensive Care Med 44:1914–1922.
2. Paudel R et al, (2021) Mechanical Power: A New Concept in Mechanical Ventilation, The American Journal of the Medical Sciences,Volume 362, Issue 6, 2021,537-545,
3. Thoral PJ, Peppink JM, Driessen RH, et al (2021) Sharing ICU Patient Data Responsibly Under the Society of Critical Care Medicine/European Society of Intensive Care Medicine Joint Data Science Collaboration: The Amsterdam University Medical Centers Database (AmsterdamUMCdb) Example. Crit Care Med 49:e563–e577.
4. Becher T, van der Staay M, Schadler D, et al (2019) Calculation of mechanical power for pressure-controlled ventilation. Intensive Care Med 45(9): 1321-1323.
5. Kacmarek RM, Villar J.. Management of refractory hypoxemia in ARDS. Minerva Anestesiol 2013;79(10):1173–1179.
6. Rees, S.E., Spadaro, S., Dalla Corte, F. et al. Transparent decision support for mechanical ventilation using visualization of clinical preferences. BioMed Eng OnLine 21, 5 (2022).

Team 6: Positive-Pressure-Pose

AI for Intensive Care: Tailoring Mechanical Ventilation and Power with Reinforcement Learning

Tariq Dam[1], Ameet Jagesar[1], Laurens Biesheuvel[1], Martijn Otten[1], Bob van Kempen[1], Jim Smithuis[1], Gabriele Mondelli[2], Michael Toolan[3], Cagdas Okay[4]

[1]Intensive Care Medicine, Amsterdam UMC, Amsterdam, Netherlands. [2]Flevoziekenhuis, Almere, Netherlands. [3]Guy's and St Thomas' Hospital, London, United Kingdom. [4]Whittington Health NHS Trust, London, United Kingdom.

Correspondence: L. Biesheuvel (l.biesheuvel@amsterdamumc.nl)

Introduction. Mechanical ventilation in Intensive Care Units (ICU) requires a delicate balance: optimising gas exchange without inducing ventilator induced lung injury (VILI). High mechanical power (MP) can exacerbate VILI and reduce ventilator-free days (VFDs) [5, 9, 10]. This study aims to develop a reinforcement learning (RL) algorithm, to fine-tune the individual components of MP directly adjustable on a ventilator, aiming to personalise ventilation settings and improve patient prognosis.

Methods. A retrospective study was conducted on AmsterdamUMCdb, a comprehensive, de-identified ICU patient database from the Amsterdam UMC in the Netherlands between 2003 and 2016. The study population included patients undergoing pressure-controlled mechanical ventilation for at least 24 hours. The data were segregated into 200 folds of 70% training and 30% test sets. Patients were clustered into 500 clusters, 'states', using k-means clustering of the features listed in Table 1. The time window for each state was set at 4 hours, the maximal length of a trajectory was truncated at 72 hours. Incorporating RL in a retrospective database necessitates a rich database of clinician actions, 'trajectories', that mirror RL agent's suggested actions. To enhance the likelihood of this alignment, and to maintain focus on the most meaningful decision regions where clinicians' choices may vary, our method innovatively refines these trajectories by omitting states based on the mean Euclidean distance (ED) between the actions within a state and its centroid. only the 50% percent of states with the highest ED were included. The action space consisted of four ventilation parameters (PEEP, FiO2, RR and driving pressure), each divided into three bins. Tabular Q-learning, as the RL algorithm was deployed. VFDs can be a proxy for VILI severity [5] and were used as the reward metric. off-policy value estimation. Final performance was evaluated by off-policy value estimation.

Results. The study involved 2289 mechanically ventilated patients. As shown in Figure 2, both the RL agent and clinician avoided high levels of driving pressures and RR, but the RL agent chose medium to high FiO2 more frequently than clinicians. On medium FiO2 levels, the RL agent usually paired this with higher PEEP and driving pressure, whereas clinicians typically opted for lower levels of PEEP and driving pressure. When the RL agent opted for higher FiO2, it usually did so with lower driving pressure. Differences were mainly observed in the chosen levels of FiO_2 and driving pressure. When evaluating performance after 200 runs, mean performance of the RL agent was 14.6 VFDs (95% CI 8.5-20.8), while the performance for the clinician was 17.3 VFDs (95% CI 16.4-18.2).

Conclusion. This study goes beyond ascertaining the association between mechanical power and outcome, by introducing a ML algorithm to individualise ventilator settings and visualising RL agent and clinicians actions The observed variation in projected VFDs underscores the need for further refinement of the RL algorithm. The authors' future work will involve incorporation of additional features and fine-tuning of hyperparameters to enhance training and evaluation, potentially revolutionising mechanical ventilation and improving patient care.

References

1. Chen S, Qiu X, Tan X, Fang Z, Jin Y. A model-based hybrid soft actor-critic deep reinforcement learning algorithm for optimal ventilator settings. Information Sciences. 2022;611:47-64.
2. Zhang K, Wang H, Du J, et al. An interpretable RL framework for pre-deployment modeling in ICU hypotension management. npj Digital Medicine. 2022;5:173.
3. Becher T, van der Staay M, Schädler D, et al. Calculation of mechanical power for pressure-controlled ventilation. Intensive Care Medicine. 2019;45:1321-1323.
4. Peine A, Hallawa A, Bickenbach J, et al. Development and validation of a reinforcement learning algorithm to dynamically optimize mechanical ventilation in critical care. npj Digital Medicine. 2021;4:32.
5. Serpa Neto A, Deliberato RO, Johnson AEW, et al. Mechanical power of ventilation is associated with mortality in critically ill patients: an analysis of patients in two observational cohorts. Intensive Care Medicine. 2018;44:1914-1922.
6. Yarnell CJ, Angriman F, Ferreyro BL, et al. Oxygenation thresholds for invasive ventilation in hypoxemic respiratory failure: a target trial emulation in two cohorts. Critical Care. 2023;27:67.
7. Chi Y, He HW, Long Y. Progress of mechanical power in the intensive care unit. Chinese Medical Journal (Engl). 2020 Sep 20;133(18):2197-2204.
8. Zheng H, Zhu J, Xie W, et al. Reinforcement learning assisted oxygen therapy for COVID-19 patients under intensive care. BMC Medical Informatics and Decision Making. 2021;21:350.
9. Gattinoni L, Tonetti T, Cressoni M, et al. Ventilator-related causes of lung injury: the mechanical power. Intensive Care Medicine. 2016;42:1567-1575.
10. Cressoni M, Gotti M, Chiurazzi C, et al. Mechanical Power and Development of Ventilator-induced Lung Injury. Anesthesiology. 2016 May;124(5):1100-8.

Demographics	Age, BMI, gender
Observations	HR, sBP, dBP, MAP, SpO_2, temperature, U/O, P/F ratio
Lab values	Albumin, aptt, base excess, bicarbonate, bilirubin, calcium, chloride, creatinine, crp, eGFR, glucose, haematocrit, haemoglobin, inr, lactate, leucocytes, magnesium, pCO_2, pH, pO_2, potassium, sodium, thrombocytes
Medications	Noradrenaline dose, NMBA drip y/n, any fluid input
Ventilator	Compliance, FiO_2, PEEP, driving pressure, tidal volume, RR, delta-PEEP

Table 1. Features in state space

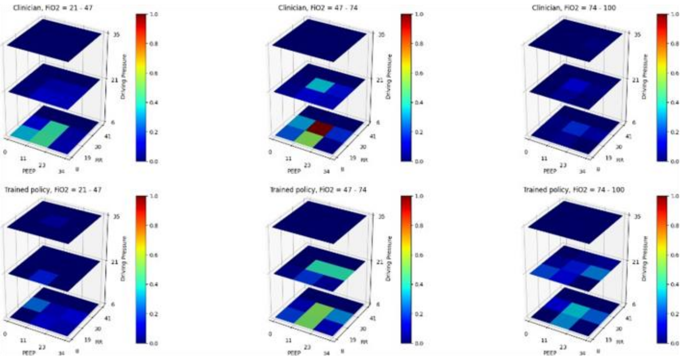

Figure 1. The heatmap compares the decisions made by clinicians (top row) with the suggestions from the RL agent (bottom row) across three different FiO2 settings. Each axis represents a specific parameter: PEEP on the X-axis, respiratory rate on the Y-axis, and driving pressure on the Z-axis. The colour coding indicates the frequency of each action's selection.

Team 7: Bed-Bytes

Simulating ICU occupancy using an anonymised ICU database

Andres Diaz-Pinto[1], Faryal Zaman[2], Marie Buckel[3], Malgorzata Starczewska[4], Vishwajit Verma[5], Abhinav Gupta[6], Vinayak Mishra[7], Jonathan O'Doherty[8], Sara Escalona Rodriguez[9], Scott Houston[10], Romit Samanta[11]

[1]King's College London, London, United Kingdom. [2]Critical Care Department, King's College Hospital, London, United Kingdom. [3]St. Bartholomew's Hospital, London, United Kingdom. [4]Royal London Hospital, London, United Kingdom. [5]Hamad Medical Corporation, Doha, Qatar. [6]Royal Free Hospital, London, United Kingdom. [7]Blackpool Teaching Hospitals NHS Foundation Trust, Blackpool, United Kingdom. [8]NHS Scotland, United Kingdom. [9]Royal Brompton and Harefield Hospital, London, United Kingdom. [10]Northern Care Alliance, Manchester, United Kingdom. [11]University of Cambridge, Cambridge, United Kingdom.

Correspondence: R. Samanta (rs307@cam.ac.uk)

Introduction. The assessment of Intensive Care Unit (ICU) capacity and the need to reliably predict occupancy in order to optimise the delivery of both elective and emergency hospital services is complex and often unreliable [1]. The 2009 H1N1 pandemic identified the need for planning of surge capacity for critical care services worldwide and how this needed reliable prediction models for critical care occupancy. More recently, the Covid-19 pandemic necessitated not only a rapid escalation of critical care capacity, but also to levels few practitioners had experienced. Several models of ICU occupancy and predictors of capacity were created to accommodate the ever-changing burden of the disease on the population [3]. As the complexity of health and disease increases, so does the demand for intensive care beds. This comes at a time when globally, there is increasing risk for both natural and man- made disasters, which could call for surge capacity at short notice. With this in mind, using data from The Amsterdam University Medical Centers Database, we built a model to predict ICU capacity to plan ordinary clinical activity as well as extraordinary surge activity.

Methods. For this analysis, we used a discrete event simulation with Poisson distributions to estimate elective and urgent patient admission rates during weekdays and weekends. The characteristics of admitted simulated patients were drawn from the UCMDb Amsterdam ICU database [4]. A multivariate distribution incorporating age group, admission SOFA score was used to generate sample patients. Admission diagnosis was used to inform patient survival and length of stay (Figure 1). Simulations were burned in for 100 days to establish stability before recording occupancy rates. Where demand exceeded capacity rejection events were recorded. A total of 1000 simulations were run to produce reliable estimates of occupancy and the frequency of rejection events. Patients with an expected longer duration of length of stay were defined as length of stay outliers and their length of stay was estimated based on the characteristics of other outlier patients in the database. We estimated ICU admissions due to seasonal variation and during the COVID-19 pandemic using the published reports from external, national data sources (NHS Digital and ICNARC, Netherlands COVID data). All analysis was performed with python using Google collab.

Results. Our simulations determined that in order to reduce rejection events less than 1 per year a capacity of 48 would be required. To maintain an occupancy of 85% the optimum ICU capacity was estimated to be 55 beds. To inform simulations under pandemic and surge conditions we used external data to estimate the increase in capacity requirements under these circumstances. We found winter months were associated with an approximate 9% increase in demand for ICU beds (figure 3). During the COVID-19 pandemic in the Netherlands, the demand for ICU beds was over 200% in the first surge, and as high as 150% in subsequent waves. (Figure 4).

References

1. Verburg IWM, Atashi A, Eslami S, et al (2017) Which Models Can I Use to Predict Adult ICU Length of Stay? A Systematic Review*. Crit Care Med. Feb;45(2):e222–31.
2. Hota S, Fried E, Burry L, Stewart TE, Christian MD (2010) Preparing your intensive care unit for the second wave of H1N1 and future surges. Critical Care Medicine Apr;38(4 Suppl):e110-9.
3. McCabe R, Kont MD, Schmit N, et al (2021) Modelling intensive care unit capacity under different epidemiological scenarios of the COVID-19 pandemic in three Western European countries. Int J Epidemiol. Jul 9;50(3):753-767.
4. Thoral PJ, Peppink JM, Driessen RH, et al (2021) Sharing ICU Patient Data Responsibly Under the Society of Critical Care Medicine/European Society of Intensive Care Medicine Joint Data Science Collaboration: The Amsterdam University Medical Centers Database (AmsterdamUMCdb) Example. Crit Care Med. Jun 1;49(6):e563-e577.

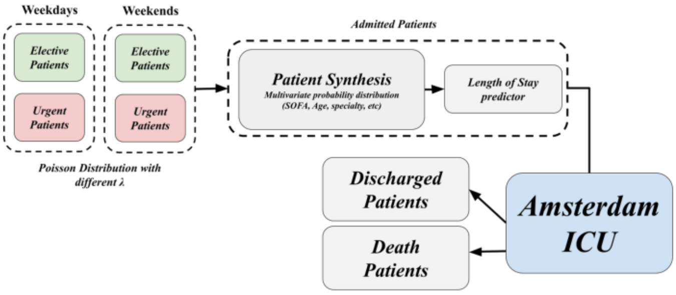

Figure 1. General schema for the simulated ICU.

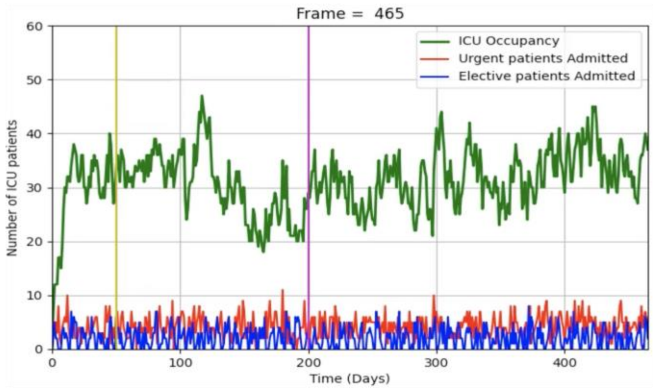

Figure 2. Simulated ICU bed occupancy for 465 days.

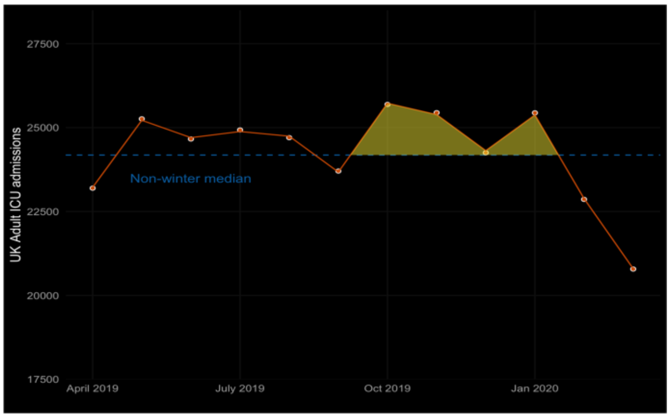

Figure 3. Seasonality in ICU admissions based on UK admission data from ICNARC. Shaded area is the excess demand over the winter months compared with the non-winter median number of admission (dashed line).

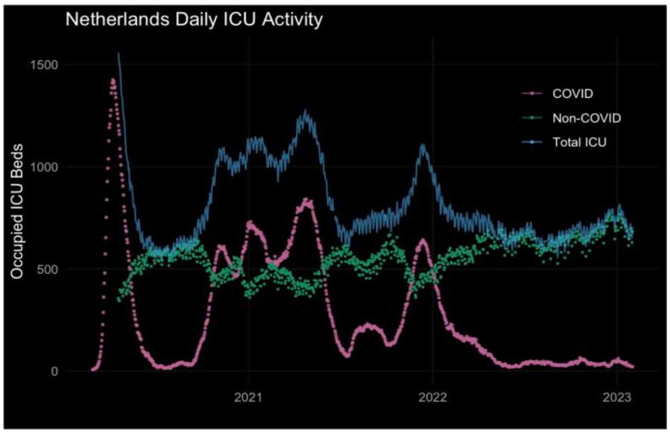

Figure 4. ICU bed occupancy during the COVID-19 pandemic for the period March 2020 to January 2023.

Team 8: ICU-Intelligence

Statistical Modelling for ICU Capacity Planning and Simulation of Pandemic Conditions on ICU Capacities based on AmsterdamUMC Database

Arash Ranjbar[1], Anastasiya Rahulina[2], Dora Karmelić[3], Ligia Iulia Torsin[4], Ema Karmelić[5]

[1]Ruder Boskovic Institute, Zagreb, Croatia. [2]Gomel Regional Clinical Hospital, Gomel, Belarus. [3]University Hospital Centre Zagreb, Zagreb, Croatia. [4]Central Military Hospital Carol Davila, Bucharest, Romania. [5]Karolinska Institutet, Stokholm, Sweden.

Correspondence: A. Rahulina (nastyo@list.ru)

Introduction. Intensive care unit (ICU) beds are a valuable and expensive resource [1]. Data science can be used to optimise ICU capacity planning and resource allocation by analyzing historical ICU data and predicting needs [2, 3, 4]. The unavailability of ICU beds was associated with increased mortality during the COVID-19 pandemic [5, 6].

Methods. We simulated ICU bed occupancy using SimPy, a Python framework, with data from the University hospital centre Amsterdam database (AmsterdamUMCdb). It included 23106 de-identified ICU patient records from 2003 to 2016. After analyzing length of stay (LOS) distribution and calculating ICU admission rate (AR) for urgent (eAR) and planned (pAR) admissions using AmsterdamUMCdb data, we simulated maximum ICU bed occupancy (MO) and generated a statistical ensemble of occupancy data by iterating the simulation. AR followed a log-normal distribution ($\sigma = 0.5$, $\mu = \text{Log}$(estimated average AR)) for both groups. pAR occurred on weekdays, eAR every day. LOS data from the dataset was used to determine LOS distribution. On each simulation day, eAR and pAR were randomly selected from the log-normal distribution and admissions were assigned based on LOS distribution. The simulation covered one year and provided daily MO. We simulated a pandemic scenario with a high number of ICU-requiring ARDS patients using the ARDS patient cohort from AmsterdamUMCdb. Their LOS were used for two simulations, both run 1000x. The first simulation was run with constant eAR of 2x, 4x and 8x of the usual eAR. The second one used the AR based on a log-normal distribution with day 21 as the pandemic's peak, when the AR reached approximately 5x the usual total AR, and the pandemic lasted for 90 days.

Results. The simulation was run 10000 times, generating an ensemble of MOs. The average MO was 23 (SD = 4.25). Considering a trimming cut-off at 90% of MO in simulations, the maximum occupancy threshold (MOT) was 28. In individual simulations, crossing MOT occurred up to 2-3 times annually (Figure 1). Aiming at {85%, 95%} occupancy rate (OR), the optimal ICU bed capacity (BC) was {33, 29}. In the ARDS pandemic simulation with the constant AR, the average MO was 15, 29 and 57 (SD= 2.30, 4.37 and 8.31, respectively) for 2x, 4x and 8x normal eAR. Given a trimming cut-off of 90%, MOTs are 19, 35 and 68, making the optimal BC {22; 41; 80}, to achieve OR of 85% respectively. For the varying AR, the average MO was 45 (SD = 2.33). Given a trimming cut-off at 90% of MO, the MOT=48. Therefore the optimal BC at 85% OR is 56 (Figure 2).

Conclusions. Within its validity range, this model can be used to predict ICU bed needs and plan resources accordingly (staffing, equipment, etc.). The model also predicts ICU requirements during a pandemic when its clinical manifestation resembles a known entity and the AR is known or assumed. Further research can enhance precision by exploring the correlation between LOS and other dataset features, enabling its use in predictive modelling.

References

1. Halpern NA, Pastores SM, Greenstein RJ. Critical care medicine in the United States 1985-2000: an analysis of bed numbers, use, and costs. Crit Care Med. 2004;32(6):1254-1259.
2. Peres IT, Hamacher S, Cyrino Oliveira FL, Bozza FA, Salluh JIF. Data-driven methodology to predict the ICU length of stay: A multicentre study of 99,492 admissions in 109 Brazilian units. Anaesth Crit Care Pain Med. 2022;41(6):101142.
3. Sanchez-Pinto LN, Luo Y, Churpek MM. Big Data and Data Science in Critical Care. Chest. 2018;154(5):1239-1248. Luo MH, Huang DL, Luo JC, Su Y, Li JK, Tu GW, Luo Z. Data science in the intensive care unit. World J Crit Care Med. 2022 Sep 9;11(5):311-316.
4. Santos AC, de Oliveira SLF, Macedo VLM, et al. Intensive Care Unit prioritization: The impact of ICU bed availability on mortality in critically ill patients who requested ICU admission in court in a Brazilian cohort. J Crit Care. 2021;66:126-131.
5. Duggal A, Mathews KS. Impact of ICU strain on outcomes. Curr Opin Crit Care. 2022;28(6):667-673.

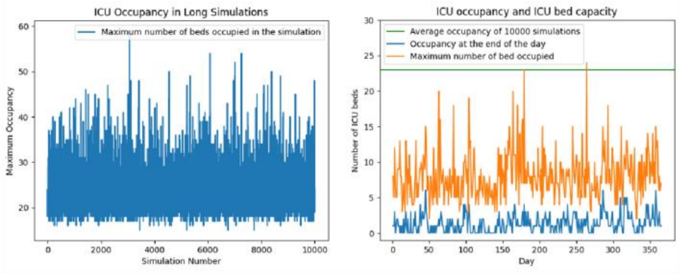

Figure 1. Maximum ICU bed occupancy simulation in 10000 repetitions and a single simulation showing ICU bed occupancy at the end of the day, daily maximum occupancy and where maximum occupancy crosses the threshold of average occupancy in 10000 simulations.

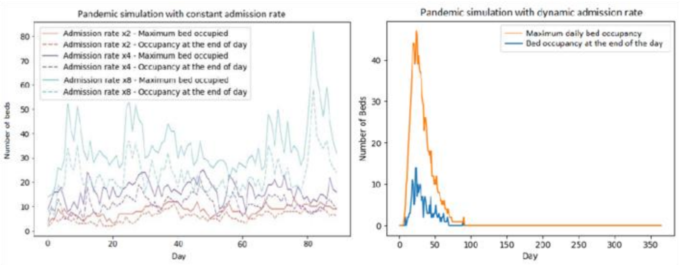

Figure 2. Simulations of ICU bed occupancy at the end of the day and maximal daily ICU bed occupancy in the simulation of a pandemic marked by spike in admission of ARDS patients to the ICU when the admission rates are constant vs. dynamical with peak at day 21.

Team 9: ACID

„With great power comes greater predictability“ - Implementation of a machine learning algorithm to predict primary weaning failure in mechanically ventilated patients

Jan Goertzen-Patin[1], Dorothea Lange[2], Clemens Grimm[3], James Hilton[4], Hanna Illian[3], Jochen Kappler[5], Asterios Tzalavras[6], Stefan Ehrentraut[5]

[1]Universitätsklinikum Bonn, Dept. of Internal Medicine, University Hospital Bonn, 53127 Bonn, Germany. [2]Department of Anesthesiology, University Hospital, LMU Munich, 81377 Munich, Germany. [3]Universitätsmedizin Göttingen, Department of Anaesthesiology, Emergency and Intensive Care Medicine, University of Göttingen, Robert-Koch-Straße 40, 37075, Göttingen, Germany. [4]Charité - Universitätsmedizin Berlin, corporate member of Freie Universität Berlin and Humboldt-Universität zu Berlin, Department of Anaesthesiology and Intensive Care Medicine, Berlin, Germany. [5]Universitätsklinikum Essen, Department of Hematology and Stem Cell Transplantation, West German Cancer Center, University Hospital Essen, 45147 Essen, Germany. [6]Universitätsklinikum Bonn, Dept. of Anesthesiology and Intensive Care Medicine, University Hospital Bonn, 53127 Bonn, Germany

Correspondance: J. Goertzen-Patin (jangoertzen@gmail.com)

Introduction. Since the rise of artificial intelligence and the increasing use of machine learning (ML) models in critical care medicine, the demand for clinical decision support systems has grown exponentially. Especially in mechanically ventilated patients, where both prolonged organ support and extubation failure pose a hazard to patient safety, identifying the "right" moment for extubation is crucial [1]. In this project, we have created a prediction model for primary weaning failure including mechanical power (MP) [2] as a measure of ventilator-induced lung injury (VILI) to predict readiness for weaning from invasive mechanical ventilation (IMV).

Method. Utilising AmsterdamUMCdb [3], we identified patients undergoing at least one episode of IMV. Restarting IMV within 24 hours of extubation was classified as primary weaning failure. In order to maintain data quality and minimize the impact of extreme outliers, our analysis was restricted to patients receiving ≤10 episodes of IMV. To predict weaning failure, we identified possible input parameters for a supervised ML algorithm. These input parameters included ventilatory parameters (e.g. PEEP, tidal volume, driving pressure), compound variables (e.g. mechanical power normalised to ideal body weight (MP/IBW), $PetCO_2/PaCO_2$, P/F-Ratio), and laboratory parameters (e.g., lowest haemoglobin (Hb), highest lactate, lowest albumin) within the last 48 hours of ventilation episodes. Feature selection was performed after identifying and removing collinear items (Pearson's correlation coefficient, threshold 0.3). An open-source machine learning library (PyCaret) was used to identify the best-performing algorithm. Using a train-test-split procedure, 30% were randomly assigned to the test cohort. K-fold cross-validation was implemented to ensure high generalisation performance (k=10). In order to counteract class imbalances, we leveraged hyperparameter tuning alongside class weights, thereby optimising the model for superior performance. We developed a Flask webapp prototype upon successful model training and testing, demonstrating our model's potential in bedside clinical decision support.

Results. We identified 15725 (32% female, median age group 60-69 years, median weight group 70-79kg) patients undergoing IMV, 918 (6.2%) of which showed weaning failure

(30% female, median age 60-69 years, median weight 80-89kg). Comparing different machine learning algorithms, the Extra Trees Classifier was identified as performing best regarding accuracy (0.97), precision (0.97), recall (0.74), and F1-score (0.84). After hyperparameter tuning and model optimisation, feature importance analysis identified lowest Hb, P/F-Ratio, MP/IBW, $PetCO_2/PaCO_2$, I:E-ratio, and gender as features with the highest values (Figure 1). The model achieved a favourable trade-off between precision and recall, minimising false positives and false negatives (Figure 2). The code for the Flask webapp has been published on GitHub (https://github.com/jangoertzen/ESICM-Webapp).

Conclusion. We have successfully created a model for predicting primary weaning failure and launched a webapp for bedside use. The model is yet to be validated using an external cohort (e.g., in a prospective multicentre study).

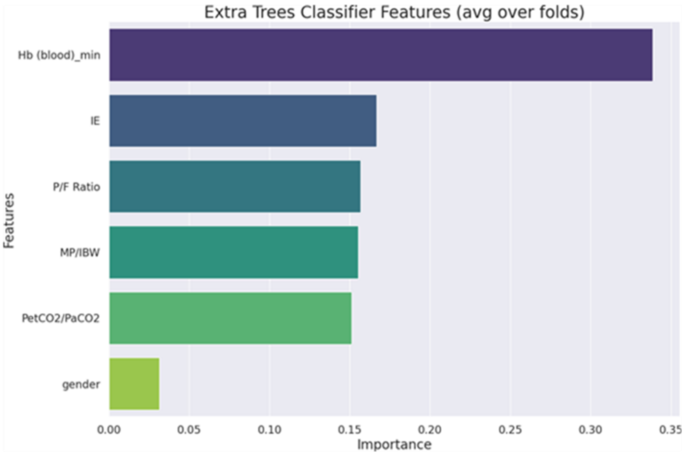

Figure 1. Feature value chart for parameters with the highest values in predicting weaning failure with the Extra Trees Classifier model.

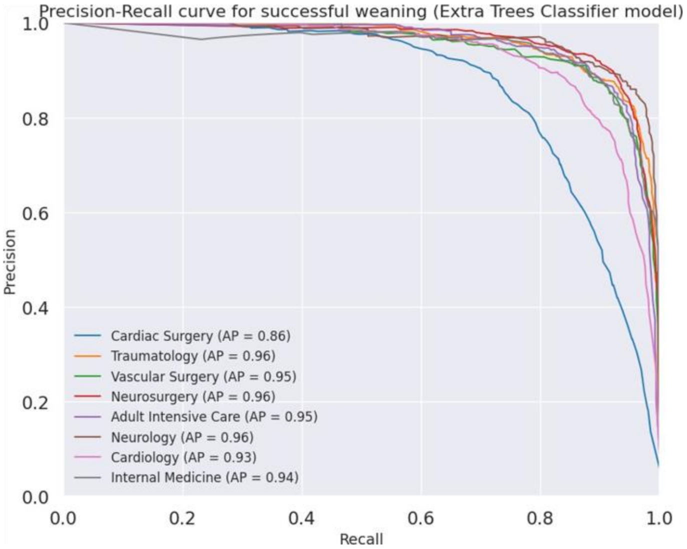

Figure 2. Precision-recall curve for prediction of weaning failure using an Extra Trees Classifier model.

References

1. Akella P, Voigt LP, Chawla S. To Wean or Not to Wean: A Practical Patient Focused Guide to Ventilator Weaning. Journal of Intensive Care Medicine. 2022;37(11):1417-1425.
2. Gattinoni L, Tonetti T, Cressoni M, Cadringher P, Herrmann P, Moerer O, Protti A, Gotti M, Chiurazzi C, Carlesso E, Chiumello D, Quintel M. Ventilator-related causes of lung injury: the mechanical power. Intensive Care Med. 2016 Oct;42(10):1567-1575.
3. Thoral PJ, Peppink JM, Driessen RH, et al (2021) Sharing ICU Patient Data Responsibly Under the Society of Critical Care Medicine/European Society of Intensive Care Medicine Joint Data Science Collaboration: The Amsterdam University Medical Centers Database (AmsterdamUMCdb) Example. Crit Care Med. Jun 1;49(6):e563-e577.

Team 10: Iberathon

Association of MP and its components with 90-day Mortality in Patients undergoing Pressure Controlled Ventilation

Alexandra Binnie[1], Daniel Pestana[1], Jesus Rico-Feijoo[2], Gabriel Moreno-Gonzalez[3], André Oliveira[4], José Cidade[5], Ana Rita Nogueira[6], Ana Claudia Cunha[7]

[1]Algarve Biomedical Centre, Faro, Portugal. [2]Surgical Critical Care Unit, Rio Hortega University Hospital, Valladolid, Spain. [3]Intensive Care Department, Hospital Universitari de Bellvitge, L'Hospitalet de Llobregat, Spain. [4]Vila Franca de Xira Hospital, Vila Franca de Xira, Portugal. [5]Centro Hospitalar de Lisboa Ocidental, Lisboa, Portugal. [6]Centro Hospitalar e Universitário de Coimbra, Coimbra, Portugal. [7]Intensive Care Unit, Centro Hospitalar de Leiria, Leiria, Portugal.

Correspondance: A. Binnie (alexandra.binnie@gmail.com)

Introduction. Invasive mechanical ventilation is life-saving for patients with acute respiratory failure (ARF); however, high airway pressures, large tidal volumes and elevated respiratory rates can provoke ventilator-induced lung injury [1], which prolongs respiratory failure and increases the risk of other organ failures and mortality [2]. Given the importance of mitigating such risks, lung-protective ventilation is central to the management of mechanically ventilated patients [3]. Mechanical Power (MP) combines pressure, volume and respiratory rate in a single variable and has emerged as a unifying variable that can quantify the energy applied to the respiratory system [4]. Previous studies have shown that MP is associated with intensive care unit (ICU) and hospital mortality [5]. However, it is unclear whether this is a direct effect of MP or a function of the underlying severity of illness. Understanding the relationship between MP and mortality would enable an individualised approach to ventilator management, potentially improving patient outcomes. In this study, we analysed a retrospective cohort of ICU patients undergoing pressure controlled ventilation (PCV) to determine the impact of MP on mortality.

Method. AmsterdamUMCdb is a publicly-accessible database of 23,106 ICU admissions in the Netherlands between 2003-2016 [6]. Patients were included if they were >18 years old, admitted to ICU for > 72 hours and received PCV. Only the 1st admission was included and patients receiving extracorporeal membrane oxygenation were excluded. MP was calculated using the simplified formula:

$$MP = 0.098 \cdot RR \cdot [Vt \cdot (\Delta P + PEEP)]$$

where RR is respiratory rate, Vt is tidal volume, **Δ**P is inspiratory pressure, and PEEP is positive end-expiratory pressure [7]. Data analysis was conducted using Google Bigquery, python and R.

Results. A total of 4201 adult surgical and medical patients were included in the analysis. MP and MP per ideal body weight (MP/IBW) were associated with mortality in patients with low DC (< 20 ml/cmH_2O) and low PaO_2/FiO_2 ratios (< 100 mmHg) but not in patients with high DC (> 40 ml/cmH_2O) and high PaO2/FiO2 ratios (> 200 mmHg). Using an iterative algorithm, we analysed the impact of MP on mortality per day of PCV. At increasing MP thresholds, patients with high or moderate DC showed only a small increase in mortality per day (Figure 2). Patients with low DC, however, showed a significant increase in mortality above 25 J/min. This effect was associated with high airway pressures and volumes but not with increased respiratory rate. Target trial emulations were conducted to measure the impact of MP and its components on ventilator-free-days at Day 28 (VFD-28) while controlling for confounding. In these emultations, only MP/IBW was associated with a decrease in VFD-28.

Conclusion. MP/IBW is strongly associated with mortality in patients with low DC but not in those with high DC. In patients with low DC, mortality increases significantly for each day of PCV above 25 J/min. In a target trial emulation. MP/IBW but not MP was associated with fewer VFD-28.

References

1. Slutsky, A. S. & Ranieri, V. M. Ventilator-induced lung injury. The New England journal of medicine 370, 980 (2014).
2. Coppola, S. et al. Effect of mechanical power on intensive care mortality in ARDS patients. Crit Care 24, 246 (2020).
3. Gattinoni, L. et al. The future of mechanical ventilation: lessons from the present and the past. Crit Care 21, 183 (2017).
4. Gattinoni, L. et al. Ventilator-related causes of lung injury: the mechanical power. Intensive Care Med 42, 1567–1575 (2016).
5. Neto, A. S. et al. Mechanical power of ventilation is associated with mortality in critically ill patients: an analysis of patients in two observational cohorts. Intensive Care Med 44, 1914–1922 (2018).
6. Thoral PJ, Peppink JM, Driessen RH, et al (2021) Sharing ICU Patient Data Responsibly Under the Society of Critical Care Medicine/European Society of Intensive Care Medicine Joint Data Science Collaboration: The Amsterdam University Medical Centers Database (AmsterdamUMCdb) Example. Crit Care Med. Jun 1;49(6):e563-e577.
7. Becher, T. et al. Calculation of mechanical power for pressure-controlled ventilation. Intensive Care Med 45, 1321–1323 (2019).

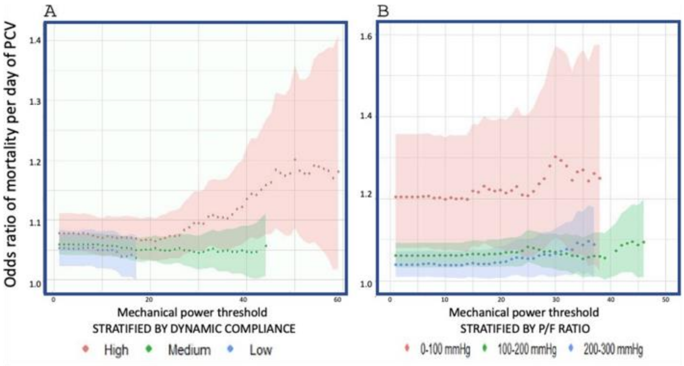

Figure 1. Odds ratio of 90-day mortality (y-axis) for each day of PCV above a given MP threshold (x-axis). Results are stratified by DC (panel A) or P/F ratio (panel B). Shading represents 95% confidence intervals.

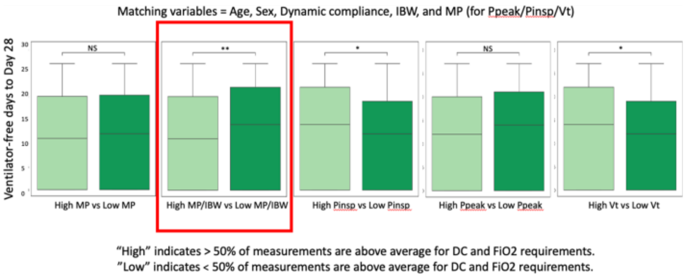

Figure 2. Target trial emulations show the relationship between high vs low MP, MP/IBW, dP, Ppeak and Vt with VFD-28 as the outcome variable. The "high" cohort comprised patients with > 50% of ventilator settings above the average for that DC and FiO_2 stratum. This was propensity score matched to a cohort of "low" patients for whom ≤50% of ventilator settings were adobe the average .

Team 12: ICU-Eliudists

The Race of Power and Pressure

Hatem Elkady[1], Bhavna Singh[2], Marilaeta Cindryani[3], Asurya Vardhan[4], Nahla Ismail[5]

[1]University of Sydney, Sydney, Australia. [2]Prof. Ngoerah Hospital, Denpasar, Indonesia. [3]Narayana Health City, Bengalaru, India. [4]Armed Forces Medical College, Wanowrie, India. [5]National University of Malaysia, Bangi, Malaysia

Correspondance H. Elkady (hatem.elkady@health.nsw.gov.au)

Introduction. Low tidal volumes, safe plateau and driving pressures (DP), and a low respiratory rate (RR) are the goals of lung protective ventilation strategies [1]. These numerical objectives, however, do not clarify the molecular origins of tissue harm. By combining the inspiratory pressure, tidal volume, and RR into a single value, mechanical power (MP) calculates the energy provided to the respiratory system. According to early findings, severe pneumonia patients who are ventilated often have higher MP, which is a reliable predictor of mortality [2]. MP was linked to 28-day mortality in ventilated patients with severe pneumonia in another cohort [3] as well it demonstrated superior predictive value for 28-day mortality compared to DP. The goal of this study is to analyse the dataset to investigate the relationship between MP and mortality in patients receiving mechanical ventilation (MV) for respiratory failure.

Methods. Using cohorts of patients from the Amsterdam University Medical Centres Database (AmsterdamUMCdb) who were admitted to the ICU and MCU from 2003 to 2016 [4], a retrospective study was conducted. All hypoxic patients > 18 years who received pressure- controlled modes of MV for more than 48 hours were included. Elective hospitalisations and post- operative cardiothoracic surgical patients were excluded. The primary outcome is the correlation between MP and ICU mortality. MP was calculated using a simplified formula [5] as time- weighted average of 6-hour block between 21 and 27 hours from initiation of MV, and the relationship between MP and mortality was investigated using logistic regression models. Other relevant variables are extracted including peak airway and plateau pressures, PEEP, inspiratory tidal volume, and respiratory rate. Other parameters were derived including DP, lung compliance and indexed tidal volume. Variables had been compared among survivors and non-survivors using appropriate statistical methods.

Results. A total of 1045 ICU admissions met the inclusion criteria, MP significantly associated with ICU mortality in patients < 60 years ($p = 0.02$) (Fig.1). Most patients were males (69.7%), median age of 61 [47-73] years, and ICU mortality was 26.3%. Median MP was 19.7 [14.3-26.7] J/min, duration of MV 7.7 [4.1-14.7] days and ICU length of stay 9.9 [5.6-18] days. DP was significantly associated with ICU mortality (OR 1.04, 95% CI 1.01-1.07, $p = 0.0039$), cut-off value for increased risk of mortality was 14.05 cmH_2O. Sensitivity analysis conducted using the level of P/F ratio showed significantly higher DP between deceased and survivors for the same level of severity of hypoxia – $PaO_2/FiO_2 < 100$ mmHg ($p = 0.037$) and significantly higher MP (for patients < 60 years) ($p = 0.029$). Multivariate regression analysis showed age and DP to be strong predictors of mortality and age, DP and RR to be predictors of ventilator-free days by day 28 (VFD_28). Using K-Means cluster unsupervised machine learning model identified 2 quite distinctive phenotypes of hypoxic respiratory failure with significant difference in mortality (19.3 vs 50%), with DP (14.1 vs 25.3 cmH_2O) and PEEP (15.4 vs 9.5 cmH_2O).

Conclusion. Higher MP is associated with an increased risk of ICU mortality in hypoxic, invasively ventilated patients less than 60 years, DP is the most important component of the MP and is associated with an increased ICU mortality and less VFD_28. Machine learning model can identify distinctive subtypes of hypoxic respiratory failure.

References

1. Fan E, Brodie D, Slutsky AS. Acute respiratory distress syndrome: advances in diagnosis and treatment. JAMA. 2018;319(7):698–710.
2. Serpa Neto A, Deliberato RO, Johnson AEW, Bos LD, Amorim P, Pereira SM, Cazati DC, Cordioli RL, Correa TD, Pollard TJ, Schettino GPP, Timenetsky KT, Celi LA, Pelosi P, Gama de Abreu M, Schultz MJ; PROVE Network Investigators. Mechanical power of ventilation is associated with mortality in critically ill patients: an analysis of patients in two observational cohorts. Intensive Care Med. 2018 Nov;44(11):1914-1922.
3. Wu H-P, Chu C-M, Chuang L-P, Lin S-W, Leu S-W, Chang K-W, Chiu L-C, Liu P-H, Kao K-C. The Association between Mechanical Power and Mortality in Patients with Pneumonia Using Pressure-Targeted Ventilation. Diagnostics. 2021; 11(10):1862.
4. Thoral PJ, Peppink JM, Driessen RH, et al (2021) Sharing ICU Patient Data Responsibly Under the Society of Critical Care Medicine/European Society of Intensive Care Medicine Joint Data Science Collaboration: The Amsterdam University Medical Centers Database (AmsterdamUMCdb) Example. Crit Care Med. Jun 1;49(6):e563-e577.
5. Trinkle, C. A., Broaddus, R. N., Sturgill, J. L., Waters, C. M., & Morris, P. E. (2022). Simple, accurate calculation of mechanical power in pressure controlled ventilation (PCV). Intensive care medicine experimental, 10(1), 22.

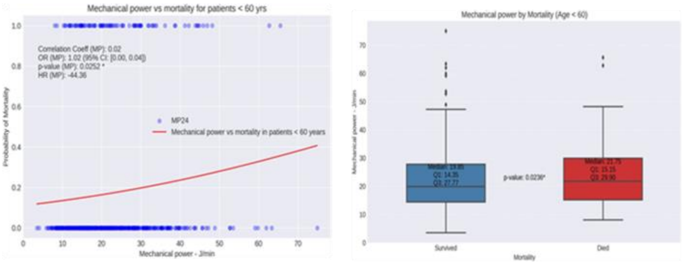

Figure 1. Logistic regression analysis of Mechanical Power vs Mortality (on the left) and box plots (on the right).

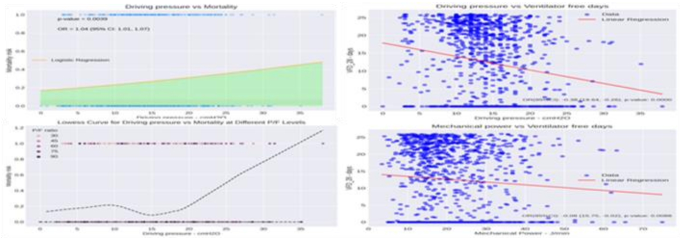

Figure 2. Driving Pressure vs mortality (left) and Mechanical Power and driving pressure (right) vs ventilator free days by day 28 (right)
